# Long-Term Survivor of Intrahepatic Cholangiocarcinoma for over 18 Years: Case Study with Longitudinal Histo-molecular and Tumor Immune Microenvironment Characterization and Systematic Review of the Literature

**DOI:** 10.1007/s12029-024-01113-8

**Published:** 2024-09-16

**Authors:** Paola Mattiolo, Mario De Bellis, Andrea Mafficini, Matteo Fassan, Michele Bevere, Calogero Ciulla, Samantha Bersani, Rita T. Lawlor, Michele Milella, Aldo Scarpa, Claudio Luchini, Andrea Ruzzenente

**Affiliations:** 1 Department of Diagnostics and Public Health, Section of Pathology, University of Verona, University and Hospital Trust of Verona, Verona, Italy; 2 Division of General and Hepato-Biliary Surgery, Department of Surgery, Dentistry, Gynecology, and Pediatrics, University of Verona, University and Hospital Trust of Verona, Verona, Italy; 3 Department of Engineering for Innovation Medicine (DIMI), University of Verona, Verona, Italy; 4 ARC-Net Research Center, University of Verona, Verona, Italy; 5 Department of Medicine (DIMED), Surgical Pathology & Cytopathology Unit, University of Padua, Padua, Italy; 6 Veneto Institute of Oncology (IOV-IRCCS), Padua, Italy

**Keywords:** Long survivor, Long survival, Cholangiocarcinoma, Intrahepatic, Biliary, Relapse

## Abstract

**Background:**

Intrahepatic cholangiocarcinoma is a biliary neoplasm usually showing a dismal prognosis. In early stages, surgical resection is the best treatment option, significantly increasing the overall survival. This approach is also recommended in the case of relapsing disease. In this study, we report the case of a patient affected by intrahepatic cholangiocarcinoma with multiple relapses and still alive for over 18 years. We also provide a systematic review regarding long-survivor (> 60 months) of intrahepatic cholangiocarcinoma.

**Case Presentation:**

A 41-year-old woman with no pathological history was diagnosed with localized intrahepatic cholangiocarcinoma and surgically treated with left hepatectomy. After the first intervention, the patients underwent three further surgical resections because of locoregional recurrences. Histologically, there were some significant similarities among all neoplasms, including the tubule-glandular architecture, but also morphological heterogeneity. The tumor immune microenvironment remained stable across the different lesions. The molecular analysis with next-generation sequencing demonstrated that all neoplasms shared the same genomic profile, including *NBN* and *NOTCH3* mutations and chromosomes 1 and 3 alterations.

**Conclusions:**

This case study highlights the essential role of a stringent follow-up after resection of intrahepatic cholangiocarcinoma for detecting early relapsing tumors. Moreover, it shows the importance of the molecular characterization of multiple tumors for understanding their real nature. The accurate study of long-surviving patients highlights the features that are critical for outcome improvement.

**Supplementary Information:**

The online version contains supplementary material available at 10.1007/s12029-024-01113-8.

## Background

Cholangiocarcinoma (CCA) is the most common primary liver cancer after hepatocellular carcinoma, accounting for 10–15% of all primary liver cancers [1–5]. CCA is an adenocarcinoma with biliary differentiation [1] that can be classified according to macroscopical appearance, histological subtype, and localization. The three main gross presentations of CCA are the periductal-infiltrating type (PI type), the mass-forming type (MF type), and intraductal growth (IG type). The PI type is characterized by tumor growth along the bile duct wall, with a typically extended perineural involvement. The MF type usually occurs as a nodular mass, associated with a better prognosis. The IG type is characterized by a polypoidal growth within the bile ducts [1, 6].

The localization of biliary malignancy plays a significant role in its prognosis and management, and it is one of the most essential categorization criteria. According to the anatomic origin along the biliary tree, intrahepatic, perihilar, and distal CCA have been recognized as distinct entities [7–9]. In particular, intrahepatic CCA (iCCA) arises from the peripheral bile ducts within the liver parenchyma, proximal to the secondary biliary ducts [1, 8–10], and accounts for up to 10–15% of all biliary tract cancers. The incidence of iCCA is steadily increasing in Western Countries [1, 11–13], reaching 1.8 and 1.09 cases per 100,000 person-years in Europe and USA, respectively [1, 2]. iCCA is typically diagnosed as an advanced disease because of the lack of specific symptoms during early stages and the absence of known risk factors [14]. The mortality rate of iCCA is high, with the 5-year overall survival ranging from 5 to 20%, and slightly higher for surgically-resected neoplasms (around 30%) [15–17].

Of note, only a tiny fraction of patients with iCCA show an unusually long survival. The study of such cases may open new interesting perspectives for a better understanding of tumor biology and clinical behavior. In this study, we focus on a 41-year-old female who received the diagnosis of iCCA. After the surgical resection, she experienced three different local relapses and as many surgical resections with the administration of adjuvant chemotherapy. To date, the patient is still alive, and the current post-surgical follow-up is 18 years (216 months), which is one of the longest according to the scientific literature. The histological analysis of the different surgical specimens showed some similarities but also morphological heterogeneity. Of note is that only the molecular characterization with next-generation sequencing could clarify that all lesions derived from the same primary. We also provide a systematic review of the literature regarding patients with iCCA showing long survival (> 60 months) recording possible similarities among these peculiar cases.

## Materials and Methods

### Case Report

As specifically detailed below, after recording a complete anamnesis and relevant clinical and radiologic examinations, the patient was hospitalized and underwent surgical intervention.

The resected specimen was sampled in the section of pathology following standardized guidelines and prepared for histological analysis with hematoxylin and eosin. The pathology report followed WHO criteria and guidelines.

Specific immunohistochemical staining was obtained per standardized procedures, as described elsewhere [18–20]. The following antibodies were tested: cytokeratin AE1/AE3 (clone: AE1-AE3, 1:100 dilution, Novocastra/UK), cytokeratin 7 (OV-TL 12/30, 1:100, Dako/Germany), cytokeratin 8/18/19 (5D3, prediluted, Leica/Germany), CEA (polyclonal rabbit antibody, 1:3000, Dako), CD10 (56C6, prediluted, Novocastra), CD56 (123C3.5, 1:500, Cell Marque/USA), Hep-Par1 (OCH1E5, 1:50, Dako), EMA (E29, 1:400, Dako), estrogen receptor (1D5, 1:100, Dako), progesterone receptor (PgR 63b, 1:150, Leica), alpha-inhibin (R1, prediluted, Leica), S100 (polyclonal rabbit antibody, 1:3000, Dako), PGP9.5 (polyclonal/rabbit, 1:200, Dako), Chromogranin-A (DAK-A3, 1:2500, Dako), Synaptophysin (27G12, prediluted, Novocastra), NSE (BBS/NC/VI-H14, 1:1000, Dako), thyroglobulin (1D4, 1:500, Novocastra), TTF-1 (8G7G3/1, 1:200, Dako), CDX2 (Cdx-2–88, 1:200 Biogenex/USA), BCL10 (331.3, 1:1000, Santa Cruz Biotechnology/USA), trypsin (polyclonal rabbit, 1:500, TEMA/Italy), and Ki67 (MIB1, 1:100, Dako).

To better investigate the biology of all neoplasms, we performed chromogenic multiplex immunohistochemistry (CM-IHC) for assessing the tumor immune microenvironment (TIME) of all samples, following standardized procedures as previously described [21, 22]. For CM-IHC, the following antibodies were tested in two different staining sets: (i) first set: CD3 (clone: LN10, source: Leica/Germany, prediluted, staining: red) and CD68 ((KP1, Dako/Germany, 1:800, DAB); (ii) second set: CD4 (clone: 4B12, source: Novocastra/UK, prediluted, DAB), CD8 (C8/144B, Dako/Germany, 1:200, red), CD20 (L26, Novocastra, prediluted, blue); (ii) third set: FoxP3 (221D/D3, Serotec/Bio-Rad/USA, 1:200, DAB), CD163 (10D6, Novocastra, 1:200, red), and CD25 (4C9, Leica/Germany, prediluted, blue). Cells were considered positive when the cell membrane was stained, with the exception of FoxP3 that was evaluated in cell nuclei. The expression of these biomarkers was assessed using a semi-quantitative (0–5) scoring system, as reported previously [23, 24]: 0 = negative (no positive cells), 1 = rare (1–10 positive cells per high-power field, 400X), 2 = low (11–20 positive cells per HPF), 3 = moderate (21–30 positive cells per HPF), 4 = high (31–50 positive cells per HPF), and 5 = very high (> 50 positive cells per HPF).

Molecular analysis has been conducted with DNA next-generation sequencing (NGS). It adopted the SureSelectXT HS CD Glasgow Cancer Core assay (www.agilent.com), hereinafter referred to as CORE, as extensively described elsewhere [25–27]. Briefly, the CORE panel for NGS spans 1.8 megabases of the genome and interrogates 174 genes for somatic mutations, copy number alterations, and structural rearrangements; the details of targeted genes are reported in Supplementary Table 1. Variants were classified following the five-tier classification system recommended by the joint consensus of the American College of Medical Genetics and Genomics and the Association for Molecular Pathology (ACMG/AMP) [28]. Variants were thus classified as benign (class 1), likely benign (class 2), variant of uncertain significance (VUS – class 3), likely pathogenic (class 4), and pathogenic (class 5).

The histological, immunohistochemical, and molecular analyses were repeated using the same procedures on the surgical specimens of all lesions.

### Systematic Review

A systematic review was performed in order to summarize the evidence regarding long-term survivors affected by cholangiocarcinoma. The systematic review adhered to the Preferred Reporting Items for Systematic Reviews and Meta-Analyses statement/guidelines, based on a preset protocol (Supplementary Table 2) [29]. Two investigators (PM and CL) independently conducted a literature search using PubMed and SCOPUS, without language restriction from database inception to August 28, 2024, for all published studies on long-term survivor patients affected by conventional cholangiocarcinoma. The following search strategy was used: [(“Long-term survivor” OR “Long term survivor” OR “long survival”) AND (“cholangiocarcinoma”)]. Exclusion criteria were (1) no original cases/original information, (2) no clinical data, (3) overall survival shorter than 60 months, (4) (peri)hilar/extrahepatic localization of the primary tumor, and (5) in vitro or animal studies.

## Results

### Case Report

#### First Neoplasm

##### Clinical History and Surgical Intervention

A 41-year-old female was admitted to the hospital for abdominal pain and weight loss of 5 kg in the last 2 months. Serum tumor markers, including alpha fetoprotein (AFP), carcinoembryonic antigen (CEA), and carbohydrate antigen 19–9 (CA19-9), showed normal levels (specific data unavailable). Abdominal ultrasound and subsequent CT confirmed the presence of an intrahepatic mass of 3.0 cm near the left hepatic duct and the left hepatic artery. The patient had no history of cancer nor of liver diseases. The biopsy was positive for carcinoma, consistent with a primary hepatic neoplasm with biliary differentiation. Thus, the patient received a left hepatectomy with locoregional lymphadenectomy and cholecystectomy.

##### Surgical Pathology

**Fig. 1:**
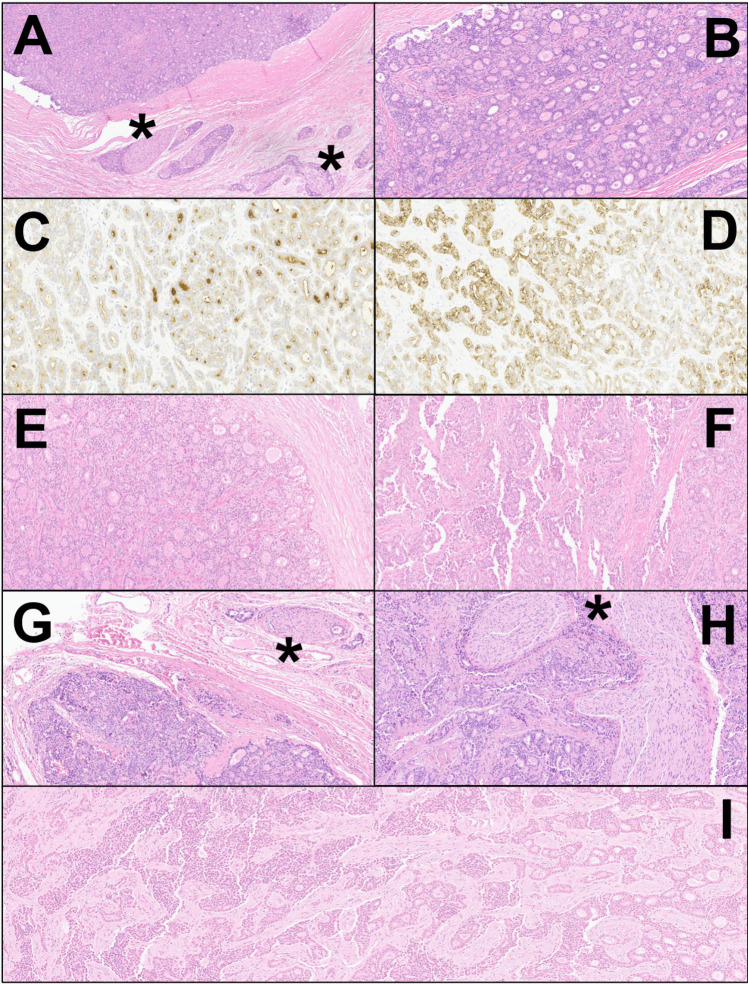
Highly illustrative figure of the most representative histological aspects of the different neoplasms here described. **A, B** First neoplasm showed tubule-glandular aggregation, with densely-packed small tubules and glands, and diffuse peri-neural infiltration (asterisks). The neoplasm was well-differentiated, hypercellular, with frequent back-to-back glands and fusions, and with areas with tubulopapillary-like patterns and cribriform features (hematoxylin–eosin, **A**: 4 × original magnification, **B**: 20x). **C, D** Immunohistochemical analysis on the first neoplasm for CEA (luminal positivity, 20x) and for cytokeratin 7 (membranous positivity, 20x). **E, F** Second neoplasm: tubule-glandular aggregation is maintained (**E** hematoxylin–eosin, 20x), but the neoplasm also showed solid pseudopapillary-like pattern (**F**: hematoxylin–eosin, 20x). **G, H** Third neoplasm: here some aspects including moderately-to-poorly differentiated small duct adenocarcinoma are evident, along with a diffuse peri-neural infiltration (asterisks) (hematoxylin–eosin, **G**: 10x, **H**: 20x). **I** Fourth neoplasm showed areas with solid and solid-trabecular architecture (left part), sometimes intermingled with areas showing tubule-glandular aggregation (right part) (hematoxylin–eosin, 10x)

Macroscopical examination revealed the presence of a 3.0 cm solid/nodular, whitish mass that centered the left hepatic lobe. The neoplasm showed tubule-glandular aggregation at histology (Fig. 1A, B), with densely-packed small tubules and glands. The neoplasm was well-differentiated and hypercellular, with frequent back-to-back glands, fusions, and areas with tubulopapillary-like patterns and cribriform features. The fibrotic stroma was present but scant. The neoplasm also showed a variable capsule, sometimes with fibrotic thickening but sometimes with infiltrative borders. At the same time, diffuse aspects of perineural infiltration were detected. Tumor cells were monomorphic and homogeneous, medium-sized, with pale eosinophilic cytoplasm and frequent perinuclear halo. Altogether, the neoplasm fell within the WHO category of well-differentiated small duct iCCA. Extralesional tissue did not show any sign of hepatic cirrhosis. All the isolated lymph nodes (*n* = 7) and the gallbladder were free from metastasis. Surgical margins were free of disease. The remaining liver parenchyma was unremarkable.

At immunohistochemistry (Fig. 1C, D, Supplementary Table 3), neoplastic cells were positive for cytokeratin AE1/AE3, 7, and 8/18/19 and showed luminal positivity for CEA. At the same time, the elements were negative CD10, CD56. Hep-Par1, estrogen and progesterone receptors, alpha-inhibin, S-100, PGP9.5, Chromogranin A, Synaptophysin, thyroglobulin, TTF-1, CDX2, BCL10, and trypsin. The Ki-67 index was comprehensively low, with rare spots reaching 7–8% of positivity. Altogether, the immunohistochemistry was consistent with the presence of a primary liver neoplasm with biliary differentiation, excluding at the same time the presence of liver metastasis from distant organs. Surgical margins were free of disease. Given the lack of multifocality and invasion of vascular structures or adjacent organs, the tumor was staged as pT1N0M0-R0, stage group I, according to the current (in 2006) TNM classification system [30].

#### Second Neoplasm

##### Clinical History and Surgical Intervention

After 5 years and 9 months, during radiologic follow-up, a liver lesion was detected. In particular, contrast-enhanced CT (CECT) demonstrated the presence of a 2.0 cm solid mass in segment seven of the liver. Of note, in this case, serum tumor markers AFP, CEA, and CA19-9 showed normal levels (AFP: 1.5 ng/mL; CEA: 1.07 ng/mL; CA 19–9: 4.65 U/mL). The patient underwent atypical resection of segment seven, aiming for radical surgery.

##### Surgical Pathology

The resected specimen presented a 2.0 cm whitish and solid nodule with well-defined margins. Histologically (Fig. 1E, F), the neoplasm showed some morphological similarities with the previously resected iCCA. In particular, this lesion also showed tubule-glandular aggregation and tubulopapillary-like and cribriform patterns, with densely-packed small tubules and glands. However, areas with ductal-plate-like aspects and solid pseudopapillary-like features were also visible at the periphery of the mass. The immunohistochemical profile was comparable to that of the previously resected tumor. Thus, based on the findings, the case was interpreted as a relapse of the previously resected iCCA. Because there was neither evidence of multifocality nor invasion of vascular structures or adjacent organs, the tumor was staged as rpT1N0M0-R0, stage group I, according to the current (in 2011) TNM classification system [31].

#### Third Neoplasm

##### Clinical History and Surgical Intervention

After 3 years and 4 months, during radiologic follow-up with CECT, there was evidence of pathologic tissue in the hepatoduodenal ligament in close contact with the right hepatic artery and common bile duct. The lesion was radiologically suspected of lymph node metastasis. Magnetic resonance (MRI) and positron emission tomography (PET) confirmed the presence of pathologic tissue. Serum tumor markers AFP, CEA, and CA19-9 values were within the normal limits (specific data unavailable). The patient underwent surgical exploration without documenting peritoneal carcinosis or liver metastasis at intraoperative ultrasound. There was only pathologic tissue in the right portion of the hepatoduodenal ligament. Considering also the young age of the patient, the poor response of iCCA to chemotherapy, and the long period free of disease, we performed the surgical resection of the common bile duct and the right hepatic artery that was infiltrated by tumor tissue, in addition to lymphadenectomy of station 8–12-13. The reconstruction phase was carried out with a Roux-en-Y biliodigestive anastomosis. The patient also received adjuvant chemotherapy (GEMOX, twelve cycles) for 6 months after surgical resection.

##### Surgical Pathology

The macroscopical examination showed the presence of a lymph node with a main axis of 3.5 cm, firm and whitish on the cut surface; the portion of the bile duct showed a length of 3.3 cm and a caliber of 0.7 cm. Subsequent histological examination (Fig. 1G, H) showed the presence of metastasis in the lymph node and tumor tissue within the bile duct wall, particularly in the muscular tunica and as a diffuse perineural tumor infiltration. Tumor morphology showed similar aspects to the previous lesions, including tubule-glandular architecture and cribriform features, but this neoplasm also showed areas with moderately-to-poorly differentiated small duct adenocarcinoma. The immunohistochemical analysis showed a very similar profile to those already observed in the previous lesions. Based on these findings, the neoplasm was interpreted as a second relapse and staged as rpT2N1M0, group IIIB, according to the current (in 2015) TNM classification system [31].

#### Fourth Neoplasm

##### Clinical History and Surgical Intervention

After 4 years and 7 months, during radiologic follow-up, CECT showed a 3 cm hypervascularized solid nodule along the hepatic resection plane and the upper margin of the duodenum with involvement of the main trunk of the portal vein and initial signs of portal hypertension. Considering the long disease-free interval, the symptomatic nature of recurrence, and the possibility of additional treatments based on chemotherapy, the patient underwent exploratory laparotomy. Serum tumor markers AFP, CEA, and CA19-9 showed normal levels (specific data unavailable). At surgery, no signs of peritoneal carcinosis or liver metastases were found; at the same time, there was evidence of tumor tissue at the distal choledochal stump with portal vein infiltration and neoplastic thrombosis. Therefore, the surgical team performed the removal of tumor tissue, tangential resection of the portal vein, portal thrombectomy, and vascular suture with a peritoneal patch. The patient also received adjuvant chemotherapy (capecitabine, six cycles) for 8 months after surgical resection but with dose reduction for acral toxicity.

##### Surgical Pathology

Because of the altered anatomy, only multiple greyish fragments ranging in length from 1 to 3.5 cm were examined and described during macroscopical evaluation. They were entirely submitted to histological examination. The tissues, including the portal vein wall, displayed a diffuse neoplastic infiltration involving nervous fascicles. Histologically (Fig. 1I), the tumor showed poorly-differentiated areas with nested and solid aggregation and focal tubule-glandular architecture. The immunohistochemical analysis showed a very similar profile to those already observed in the previous lesions. Based on these findings, the neoplasm was interpreted as a third relapse. The mass was staged yrpT2N0M0-Rx, stage group II, according to the current (2019) TNM classification system [32].

##### Current Situation

After 1 year, the patient showed radiological evidence of multifocal disease progression despite the negative tumoral markers (AFP < 3 ng/mL, CEA 2.1 ng/mL, CA19-9 12 U/mL). Thus, she received different schemes of chemotherapy, including GEMOX (interrupted for allergic reactions), FOLFIRI and irinotecan (interrupted due to liver toxicity), adriblastin (six cycles), and subsequent cisplatin and gemcitabine (eleven cycles), currently with stable disease and in maintenance with three-weekly administrations of cisplatin.

Representative radiological images, when available, have been collected and presented in Supplementary Fig. 1.

### TIME

The evaluation of CM-IHC in the different neoplasms demonstrated that the TIME was enriched in CD68 + /CD163 + tumor-associated macrophages and in CD3 + /CD8 + tumor-infiltrating lymphocytes. Tumor periphery was enriched in CD20 + lymphocytes. In particular, the following scores have been observed: CD3 (first neoplasm: 4; second neoplasm: 4; third neoplasm: 4; fourth neoplasm: 3; mean: 3.75), CD4 (2; 2; 2; 1; mean: 1.75), CD8 (3; 3; 3; 2; mean: 2.75), CD20 (intratumoral: 1; 2; 1; 2; mean: 1.5; tumor periphery: mean: 4; 4; 4; 3; mean: 3.75), CD68 (4; 4; 4; 4; mean: 4), FoxP3 (0; 1; 0; 1; mean: 0.5), CD163 (4; 4; 4; 4; mean: 4), and CD25 (1; 1; 1; 0; mean: 0.75).

### Molecular Report

Following the third relapse, considering tumor histology and the availability of new technologies for molecular analysis, NGS was performed on tumor tissues from all neoplasms (Table 1). All tumors were microsatellite stable and had a low tumor mutation burden (ranging from 1.6 to 3.2 mut/Mb). Molecular analysis was also able to show that all neoplasms shared the same genomic profile. It includes two variants of uncertain significance (VUS) of *NBN* (D129E) and *NOTCH3* (G1414V) genes and the same chromosomal alterations, as follows: loss of heterozygosity (LOH) of chromosome 1p36.33-p31.3 and chromosome 3p26.3-p14.3, and gain (4 copies) of chromosome 1q. These results definitively demonstrated that the patient suffered from the same tumor with metachronous relapses. No genetic drivers were detected.

**Table 1:** Summarizing table of the molecular alterations detected in the primitive neoplasm and its three relapses

DX	MSI	TMB	Gene alterations				Chromosomal alterations
			**Gene**	**Variation**	**VAF**	**Class**	
iCCA primitive	MSS	1.6	*NBN* *NOTCH3*	D129E G1414V	50 37	3 3	LOH chr1p36.33-p31.3, chr3p26.3-p14.3 Gain chr1q (4 copies)
iCCA first relapse	MSS	3.2	*NBN* *NOTCH3*	D129E G1414V	44 42	3 3	LOH chr1p36.33-p31.3, chr3p26.3-p14.3 Gain chr1q (4 copies)
iCCA second relapse	MSS	3.2	*NBN* *NOTCH3*	D129E G1414V	43 38	3 3	LOH chr1p36.33-p31.3, chr3p26.3-p14.3 Gain chr1q (4 copies)
iCCA third relpase	MSS	2.7	*NBN* *NOTCH3*	D129E G1414V	38 30	3 3	LOH chr1p36.33-p31.3, chr3p26.3-p14.3 Gain chr1q (4 copies)

Abbreviations: *iCCA*, intrahepatic cholangiocarcinoma; *MSI*, determination of microsatellite instability; *MSS*, microsatellite stability; *VAF*, variant allele frequency; *LOH*, loss of heterozygosity; *chr*, chromosome

### Search Results

After retrieving all potentially eligible papers from the literature using the search strategy and applying the inclusion and exclusion criteria, a total of 31 original studies were included in the systematic review (Supplementary Fig. 2) [33–63].

### Characteristics of Literature Cases

The clinicopathological data are summarized in Table 2 and reported in extenso in Supplementary Table 4. Globally considered, we found 70 cases of patients that survived for at least 60 months after receiving the diagnosis of intrahepatic cholangiocarcinoma. The patients had a slight male predominance (male–female ratio 1.28). Surprisingly, a not negligible portion of long-term survivors with iCCA have been diagnosed already at late stages. Although the absence of any information regarding tumor staging for about one-third of the cases, we reported that localized disease was observed in one-third of the cases (in particular, stage I, about 6%, and stage II, about 29%). About 10% of the patients presented with infiltration of the peritoneum or adjacent structures, while lymph nodal or distant metastases were present in about 13% and 10% of the cases at the time of diagnosis. The overall survival ranged from 61 to 380 months, with only two cases exceeding our patient’s present survival. Only seven patients did not experience the recurrence of the disease, while the vast majority (about 80%) needed multiple treatments to handle the recurrences. A common aspect emerging from the literature is that all patients with iCCA and long survival have been treated with surgery (Table 2), further emphasizing the importance of timely surgical-based approaches for treating iCCA. The majority of patients were submitted to surgery alone; in about one-third of the cases, adjuvant chemotherapy was chosen because of the presumed high risk of progression.

**Table 2:** Summarizing table of the mean results obtained from the most important studies with long-term survivors of intrahepatic cholangiocarcinoma

Sex	Age	Staging	Histology	Grade	Management	Recurr	FU
F 25 (35.7%)	60.2	IA 1 (1.5%)	iCCA NOS 49 (70%)	G1 10 (14.3%)	S 36 (51.4%)	y 56 (80%)	108.2 [σ: 102.1–114.24]
M 32 (45.7%)	[σ: 58.7–61.7]	IB 3 (4.2%)	MF iCCA 16 (22.9%)	G2 15 (21.4%)	S + aK 18 (25.7%)	n 7 (10%)	
NA 13 (18.6%)		II 20 (28.6%)	PI iCCA 2 (2.8%)	G3 4 (5.7%)	S + K 5 (7.1%)	NA 7 (10%)	
		IIIA 2 (2.8%)	IG iCCA 1 (1.5%)	NA 41 (58.6%)	S + O 3 (4.2%)		96†
		IIIB 13 (18.6%)	CholangioloCA 2 (2.8%)		K + S + aK 1 (1.5%)		
		IV 7 (10%)			S + T 1 (1.5%)		
		NA 24 (34.3%)			NA 5 (7.1%)		

Note: Patients whose follow-up exceeds 5 years (60 months) are considered “long survival.” The reported age is the patient’s age at the diagnosis; follow-up is measured in months. Staging is based on the 8th edition of the AJCC classification. In cases in which the authors did not provide all the necessary parameters, the authors decided to report “NA.”

Abbreviations: *Recurr.*, presence of recurrence (*y*, yes; *n*, no; *NA*, not available); *FU*, follow up (months); *F*, female; *σ*, standard deviation; *iCCA*, intrahepatic cholangiocarcinoma; *NOS*, not otherwise specified; *G1*, well-differentiated; *S*, surgical treatment; *y*, presence of recurrence; *M*, male; *MF*, mass-forming pattern of growth; *G2*, moderately differentiated; *aK*, adjuvant chemotherapy; *n*, no evidence of recurrence; *NA*, value not assessed; *PI*, periductal pattern of growth; *G3*, scarcely differentiated; *IG*, intra-ductal pattern of growth; *O*, other kind of regional treatment like transhepatic arterial chemo-embolization or radiofrequency ablation; *cholangioloCA*, cholangiolocarcinoma; *K*, chemotherapy; *T*, liver transplantation; *σ*, standard deviation; *†*, median of the follow-up values

Unfortunately, little histological information was provided: in about 23% of the cases, authors documented that the lesion fell in the definition of mass-forming intrahepatic cholangiocarcinoma. The periductal infiltrating and intraductal growths were reported in about 3% and 1.5% of cases, respectively. Moreover, two cases of cholangiolocarcinoma were described. Most cases (about 21%) were graded as moderately differentiated, while well-differentiation was reported in about 14%. Three iCCAs (accounting for about 6%) presented with high-grade features.

## Discussion and Conclusions

In this study, we provide an integrative characterization of a long-term survivor of iCCA for over 18 years, one of the longest in scientific literature. After a first diagnosis of localized intrahepatic cholangiocarcinoma, the patient was surgically treated with left hepatectomy. After the first, the patients underwent three further surgical interventions because of locoregional recurrences. The fourth recurrence of the disease presented after 12 months as multifocal progression on CECT, treated multiple times with different chemotherapeutical protocols. Nowadays, after 4 years, the patient is still alive and in ongoing observation. Serum tumoral markers were tracked, but all results were negative. Histologically, there were striking similarities among all neoplasms, as well as morphological heterogeneity. The tumor immune microenvironment remained stable across the different lesions. The molecular analysis showed that all neoplasms shared the same genomic profile, demonstrating their mutual correlation. This case study has some critical implications, also highlighting some important points: (i) the essential role of a stringent follow-up after iCCA resection for detecting early relapsing tumors and for allowing surgical intervention with radical intent, (ii) showing the importance of the histo-molecular characterization of multiple tumors for a better understanding of their biology and real nature, and (iii) providing an in-depth characterization of all lesions of an iCCA long-survivor, as of their clinical/surgical management.

iCCA is considered potentially resectable if its surgical removal with negative histologic margins (R0) is feasible and, concurrently, a sufficient liver remnant can be maintained [64–69]. Surgical resection with radical intent is indeed considered the gold standard for treating cholangiocarcinoma [15, 64–69], as described in our patient. Adjuvant therapies also play a critical role based on current evidence and guidelines [66, 69–73]. Unfortunately, up to 80% of iCCA is diagnosed when they are already in advanced stages and not amenable to surgical intervention [65]. Other therapeutic solutions have been proposed in this setting, including ablation, stereotactic radiotherapy, or intra-arterial therapies [68, 74].

It is also important to note that iCCA can recur even after an R0 surgical resection, presenting as a localized disease or with widespread metastasization patterns [75]. The main site of first recurrence is intrahepatic, followed by peritoneal (locoregional). As observed in our patient, localized relapses in the early stages can be treated with surgical resections. This approach represents the best therapeutic solution in this clinical scenario [75–80], with a prognosis comparable to that of primary resections [79, 80]. The importance of surgery with radical intent, even for recurrent tumors, highlights the need for strict follow-up also for patients with R0 surgical resections. In the case reported here, the strict radiological follow-up was instrumental in guaranteeing the possibility of surgical approaches to three different relapses and, ultimately, the long survival. Although standard follow-up schedule has yet to be written because of the lack of survival and cost-effectiveness data, current European Society of Medical Oncology (ESMO) indications suggest visiting patients every 3–6 months during the first 2 years after the first line of treatment. The appointments should include clinical evaluation, blood tests, and radiological imaging (preferably CECT) [4]. In cases of diagnostic doubts, PET imaging should investigate suspected relapse, and treatment decisions should be discussed with a multidisciplinary team in the presence of surgeons, pathologists, oncologists, hepatologists, radiologists, and radiotherapists. It is essential to acknowledge that the clinical setting in cases of recurrent iCCA is typically very complex. Frequently, recurred neoplasms are more aggressive and highly invasive, and the potentially remaining volume of the functioning liver after resection can represent a critical limitation to this type of approach [74]. Current scientific evidence indicates surgical resection as the first choice of treatment of iCCA relapses, especially in the following cases: (i) single site and small recurrence, (ii) negative lymph-node metastases at previous surgery, (iii) possibility of surgery preserving adequate liver function, (iv) long disease-free interval, and (v) good patient performance status; for patients who are not fit for surgery, different combinations of multimodal therapies, including systemic and local treatments and also immunotherapy, have been proposed [72, 73, 81, 82]. In the favorable cases of long-term survival, follow-up indications still need to be implemented. Although the appointment scheduling should consider multiple variables and, again, the multidisciplinary evaluation is mandatory, ESMO guidelines suggest the possibility of a lifelong screening [4]. Therefore, treatment in highly specialized centers and multidisciplinary management is critical.

One of the most significant peculiarities of the case here presented is the long survival (> 18 years). It is one of the longest in the scientific literature, as highlighted also by a systematic literature review on this topic. The first consideration is that a limited number of long-term survivors have been reported in all-time literature (70 patients). Thus, even considering that multiple cases have not been documented, we acknowledge that these cases are outstandingly uncommon and represent an exception to the usual course of iCCA progression. Among the 70 patients, there was a slight male predominance. Localized disease was present in about 30% of cases, while a non-negligible fraction (about 23%) had metastatic disease at the time of diagnosis. Among all long-term survivors, the overall survival ranged from 61 to 380 months, with only two cases exceeding our patient’s survival. Moreover, a common aspect emerging from the literature is that all patients with iCCA and long-term survival have been treated with surgery (Table 2), further emphasizing the importance of timely surgical-based approaches for treating iCCA. The majority of patients were submitted to surgery alone; one-third of the patients received adjuvant chemotherapy because of the presumed high risk of progression. The high variability in the clinical presentation of patients and the algorithms for treatment decisions represent critical challenges for finding robust prognosticators in the context of long-term survivors. At the same time, it is noteworthy to highlight that the vast majority (about 80%) of cases recurred, and this result further points out the importance of a strict follow-up in surgically resected patients with iCCA. Information about serum tumoral markers was reported for fourteen patients of the systematic review. However, their potential value for early recurrence diagnosis appeared limited in such cases. Indeed, if some cases presented a serum tumoral marker increase [36, 40, 45, 51], the positive result never anticipated the symptoms’ occurrence or the relapse on radiological images. However, the literature suggests that markers such as CA 19–9 could play a major role as prognostic moderator, identifying those patients that would benefit from a stricter follow-up [4, 83–85].

This case study and the literature review represented an opportunity to highlight the heterogeneous biological behavior of iCCA. This aspect calls for implementing new strategies for supporting clinical decisions and prognostication, ideally identifying those cases with aggressive course vs. those with more indolent behavior. Several studies have tried to recognize potentially negative prognostic moderators in recent years. Among them, some of the most reliable can be detected with biochemical blood analysis and include low albumin serum level [86–88], high neutrophil-to-lymphocyte ratio [86, 87], high platelet count [88], high CA 19–9 serum level [86, 87, 89, 90], and high CEA serum level [84, 88–90]. Other parameters, tumor-related and with poor prognostic significance, have been reported and include large tumor size [86, 87], nodal involvement [4, 91], vascular invasion [4, 91, 92], poorly differentiation [92], presence of satellite lesions [78], periductal-infiltrating pattern [93], and early recurrence after surgery [78, 89, 94]. Conversely, cholangiolocellular histotype and small duct histology [1, 93–96], high density of tumor-infiltrating lymphocytes [97], and the mass forming pattern and [1, 97] usually show better overall survival. Along those lines, recent advances call for a role for artificial intelligence in this setting [98–100]. The last development along this line is pointed out by a recent study, which showed that artificial intelligence optimal survival tree (OPT) identified subgroups within iCCA relative to long-term outcomes [85]. This OPT-based approach indicated that different margin widths based on patient and disease characteristics may optimize iCCA long-term survival [85].

Some considerations should be made in the histo-molecular investigations. All lesions developed by the patient described here showed some histo-morphological similarities, including the tubule-glandular architecture and well-differentiated areas with tubulopapillary-like and cribriform patterns. However, there were also striking differences, including the focal pseudopapillary-like features observed in the first relapse, the moderately-to-poorly differentiated small duct adenocarcinoma of the second relapse, and the solid aggregation observed in the last relapse. In this complex histological scenario, the molecular analysis showed that all lesions shared the same genomic profile, definitively demonstrating that the patient suffered from one primary tumor with multiple/metachronous relapses. Of note, molecular analysis of iCCA has currently entered clinical practice due to its enrichment in actionable alterations, including *IDH1*/*2* variations, *HER2* amplifications, and *FGFR*-genes rearrangements/fusions [101–104]. In this case, no genetic drivers have been detected. However, the molecular characterization was instrumental in understanding tumor evolution and the real nature of all neoplasms since the presence of the same molecular alterations represents solid proof of clonality, even though 69 months passed before the first relapse. *NBN* and *NOTCH3* genes have been recognized as critical genetic drivers in a fraction of cholangiocarcinomas [105–108]. Regarding *NBN*, the association between DNA double-strand-breaks-repair gene mutations and cancer development relies on the predisposition of the mutated cell to gain multiple genetic errors [109–111]. Regarding *NOTCH* and its pathway, their alterations appear to be related specifically to cholangiocarcinoma. Indeed, such pathway is physiologically activated and responsible for liver differentiation during the fetal period. In adults, the Notch-mediated conversion of the hepatocyte would be responsible for the development of intrahepatic tumors with biliary phenotype [112–114]. Experimental studies demonstrated the efficacy of Notch-tailored therapies in targeting neoplastic cholangiocytes and cancer-associated fibroblasts [115–117]. The variations of *NBN* and *NOTCH3* genes in our study have been classified as variants with uncertain significance; thus, they cannot be used for designing therapeutic strategies for precision oncology. Further studies are needed to investigate the potential clinical value of the specific reported mutations.

Interestingly, the immune tumor microenvironment remained stable across the different neoplasms, with the same or very similar immunohistochemical scores for the different cell populations. Overall, the TIME could be interpreted as immunogenic. Indeed, although TIME was enriched in tumor-associated macrophages, which can for a barrier that shields tumor cells from immune surveillance [118], it was also enriched in CD3 + /CD8 + tumor-infiltrating lymphocytes, a feature that is usually associated with prolonged survival [118].

The low frequency of iCCA with prolonged survival is an intrinsic limitation of this topic, including the current research, and it is magnified by the lack of systematic descriptions of patients with similar survival indices in the literature. In the complex clinical scenario of iCCA, where the vast majority of patients die from the disease within 5 years from the diagnosis, we would highlight three essential messages: (i) the importance of a strict follow-up for surgically-resected patients: it is the only way to detect early relapses, potentially allowing a re-intervention; (ii) the importance of surgical treatments, even in compromised anatomy and including repeated surgery aiming at radical resection; (iii) the use of histo-molecular analysis to better understand tumor biology and evolution.

In conclusion, this study presents a long survivor of iCCA and also discusses the main findings compared to the existing literature. This case highlights the essential role of a stringent follow-up after iCCA resection, the benefit of repeated surgery aiming to radical resection, and the importance of the histo-molecular characterization of multiple tumors. Understanding in depth the biology of long-survivor iCCA may represent a critical step in advancing diagnostic and therapeutic strategies for this tumor type.

## Supplementary Information

Supplementary Fig. 1 Collection of representative radiological images (CT, computed tomography), when available, of the patient during the years. A. Second relapse, CT scan (arterial phase; RHA: right hepatic artery, CBD: common bile duct); please note that the red asterisk indicates tumor recurrence. B. Second relapse, CT scan (venous phase; LPV: left portal vein, PV: portal vein); please note that the red asterisk indicates tumor recurrence. C. Third relapse, CT scan (arterial phase); please note that the red circle indicates the neoplastic thrombosis of the portal vein. D. Current situation, CT scan (arterial phase). The image clearly shows the multifocal disease progression into the liver (multiple nodules). (PNG 1463 kb)

High resolution image (TIFF 8549 kb)

Supplemental Fig. 2 (DOCX 46 kb)

Supplemental Table 1 (PDF 42 kb)

Supplemental Table 2 (DOCX 29 kb)

Supplemental Table 3 (DOCX 16 kb)

Supplemental Table 4 (DOCX 37 kb)

## Data Availability

All data are available in the manuscripts, tables, and supplementary files.

## List of Abbreviations

CCA: Cholangiocarcinoma
PI: Periductal-infiltrating
MF: Mass-forming
IG: Intraductal growth
iCCA: Intrahepatic cholangiocarcinoma
WHO: World Health Organization
NGS: Next-generation sequencing
ACMG/AMP: American College of Medical Genetics and Genomics and the Association for Molecular Pathology
TIME: Tumor immune microenvironment
AFP: Alpha-fetoprotein
CEA: Carcinoembryonic antigen
CA 19-9: Carbohydrate antigen 19–9
CT: Computed tomography
CECT: Contrast-enhanced computed tomography
MRI: Magnetic resonance imaging
PET: Positron emission tomography
TNM: Tumor node metastasis
VUS: Variants of uncertain significance
