# Supplementary figures and images for: Long-Term Survivor of Intrahepatic Cholangiocarcinoma for over 18 Years: Case Study with Longitudinal Histo-molecular and Tumor Immune Microenvironment Characterization and Systematic Review of the Literature
Source: J Gastrointest Cancer. 2024 Sep 16;55(4):1634–46. doi: 10.1007/s12029-024-01113-8 (PMC11464565; doi:10.1007/s12029-024-01113-8)

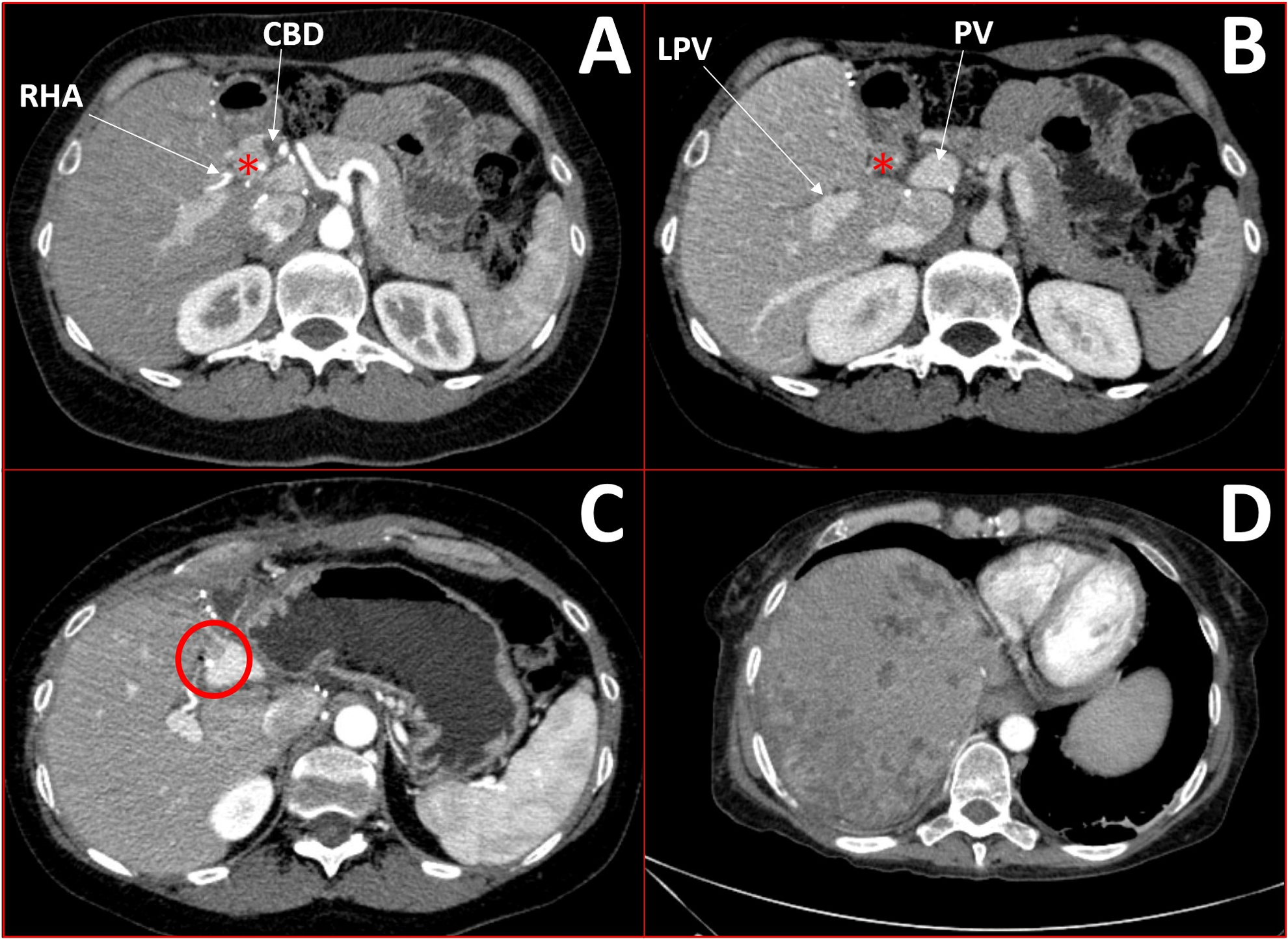

Supplement: Supplementary file 1 — Collection of representative radiological images (CT, computed tomography), when available, of the patient during the years. A. Second relapse, CT scan (arterial phase; RHA: right hepatic artery, CBD: common bile duct); please note that the red asterisk indicates tumor recurrence. B. Second relapse, CT scan (venous phase; LPV: left portal vein, PV: portal vein); please note that the red asterisk indicates tumor recurrence. C. Third relapse, CT scan (arterial phase); please note that the red circle indicates the neoplastic thrombosis of the portal vein. D. Current situation, CT scan (arterial phase). The image clearly shows the multifocal disease progression into the liver (multiple nodules). (PNG 1463 kb) [file 12029_2024_1113_Fig2_ESM.png]

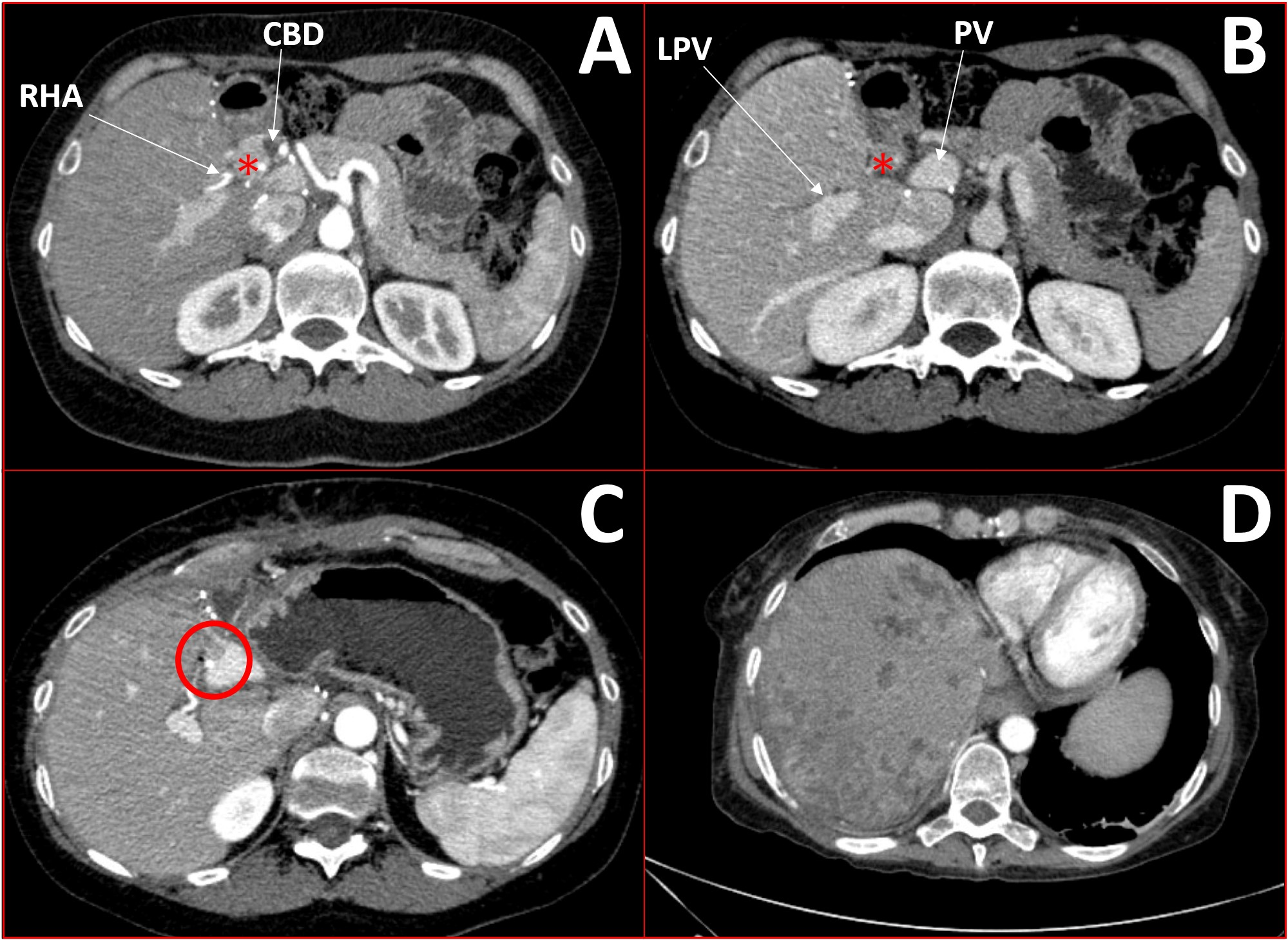

Supplement: Supplementary file 2 — High resolution image (TIFF 8549 kb) [file 12029_2024_1113_MOESM1_ESM.tiff]
