# Supplementary material for: Long-Term Survivor of Intrahepatic Cholangiocarcinoma for over 18 Years: Case Study with Longitudinal Histo-molecular and Tumor Immune Microenvironment Characterization and Systematic Review of the Literature
Source: J Gastrointest Cancer. 2024 Sep 16;55(4):1634–46. doi: 10.1007/s12029-024-01113-8 (PMC11464565; doi:10.1007/s12029-024-01113-8)
Supplement: Supplementary file 3 — (DOCX 46 kb) [file 12029_2024_1113_MOESM2_ESM.docx]

**Supplementary Figure 2. PRISMA diagram for this systematic review.**

Records after duplicates removed
(n = 64)

Additional records identified through other sources
(n = 24)

Records identified through database searching
(n = 40)

## Identification

## Screening

Records screened by title and abstract
(n = 64)

Records excluded
(n = 0)

Full-text articles excluded, with reasons
(n = 33)

*Hilar/Distal CCA (n=11)*

*No clinical data (n=5)*

*No long-term survivors (n=8)*

*Variants of CCA (n=8)*

*No human study (n=1)*

## Eligibility

Full-text articles assessed for eligibility
(n = 64)

Studies included in quantitative synthesis (systematic review)
(n = 31)

## Included

Abbreviations: CCA: cholangiocarcinoma
