# Supplementary material for: Long-Term Survivor of Intrahepatic Cholangiocarcinoma for over 18 Years: Case Study with Longitudinal Histo-molecular and Tumor Immune Microenvironment Characterization and Systematic Review of the Literature
Source: J Gastrointest Cancer. 2024 Sep 16;55(4):1634–46. doi: 10.1007/s12029-024-01113-8 (PMC11464565; doi:10.1007/s12029-024-01113-8)
Supplement: Supplementary file 6 — (DOCX 16 kb) [file 12029_2024_1113_MOESM5_ESM.docx]

|  | **First neoplasm** | **Second neoplasm** | **Third neoplasm** | **Fourth neoplasm** |
| --- | --- | --- | --- | --- |
| **CKAE1-A3** | Pos | Pos | Pos | Pos |
| **CK8-18-19** | Pos | Pos | Pos | Pos |
| **CK7** | Pos | Pos | Pos | Pos |
| **pCEA** | Pos-L | Pos-L | Pos-L | Pos-L |
| **CD56** | Neg | Neg | Neg | Neg |
| **Hep-Par1** | Neg | Neg | Neg | Neg |
| **ER** | Neg | Neg | Neg | Neg |
| **PR** | Neg | Neg | Neg | Neg |
| **Alpha-inhibin** | Neg | Neg | Neg | Neg |
| **S-100** | Neg | Neg | Neg | Neg |
| **PGP9.5** | Neg | Neg | Neg | Neg |
| **Chromogranin-A** | Neg | Neg | Neg | Neg |
| **Synaptophysin** | Neg | Neg | Neg | Neg |
| **NSE** | Neg | Neg | Neg | Neg |
| **Tg** | Neg | Neg | Neg | Neg |
| **TTF-1** | Neg | Neg | Neg | Neg |
| **CDX2** | Neg | Neg | Neg | Neg |
| **BCL10** | Neg | Neg | Neg | Neg |
| **Trypsin** | Neg | Neg | Neg | Neg |
| **GATA-3** | NA | Neg | Neg | Neg |
| **PAX-8** | NA | Neg | NA | Neg |
| **Ki-67%** | 7-8% | NA | NA | NA |

**Supplementary Table 3**. Summarizing table of all immunohistochemical markers tested in the different lesions, with results.

**Abbreviations**. Neg: the test resulted negative, Pos: the test resulted positive, Pos-L: the test resulted positive only in luminal portion of the neoplastic cells, NA: not assessed.
