# Supplementary material for: Long-Term Survivor of Intrahepatic Cholangiocarcinoma for over 18 Years: Case Study with Longitudinal Histo-molecular and Tumor Immune Microenvironment Characterization and Systematic Review of the Literature
Source: J Gastrointest Cancer. 2024 Sep 16;55(4):1634–46. doi: 10.1007/s12029-024-01113-8 (PMC11464565; doi:10.1007/s12029-024-01113-8)
Supplement: Supplementary file 7 — (DOCX 37 kb) [file 12029_2024_1113_MOESM6_ESM.docx]

**Supplementary Table 4**. Summarizing table of the clinical and pathological data obtained from the systematic review studies.

| First author | Year | Sex | Age | TNM | Histology | Grade | Management | Recurr. | FU |
| --- | --- | --- | --- | --- | --- | --- | --- | --- | --- |
| Horie^33^ | 1995 | M | 40 | NA | iCCA NOS | G1 | S | n | 144 |
| Shimahara^34^ | 1998 | F | 57 | T4N0M0 | iCCA NOS | G2 | S + aK | n | 114 |
| Nozaki^35^ | 1998 | M | 58 | T3N1M0 | MF iCCA | NA | S | NA | 68 |
| Saiura^36^ | 1999 | F | 67 | T1aN1M0 | iCCA NOS | G1 | S | y | 120 |
| Murakami^37^ | 2000 | F | 64 | NA | PI iCCA | G2 | S | n | 84 |
| Suzuki^38^ | 2002 | NA | NA | T3N0M0 | MF iCCA | NA | S | y | 131 |
| Suzuki^38^ | 2002 | NA | NA | T2N0M0 | MF iCCA | NA | S | y | 121 |
| Suzuki^38^ | 2002 | NA | NA | T2N1M1 | MF iCCA | NA | S | y | 82 |
| Suzuki^38^ | 2002 | NA | NA | T2N1M1 | MF iCCA | NA | S | y | 75 |
| Yamamoto^39^ | 2002 | M | 50 | T1aN1M1 | iCCA NOS | G2 | S | n | 96 |
| Kurosaki^40^ | 2005 | M | 74 | T2N0M0 | MF iCCA | NA | S | n | 72 |
| Kurosaki^40^ | 2005 | F | 71 | T4N0M0 | PI iCCA | NA | S + aK | y | 96 |
| Akatsu^41^ | 2005 | F | 76 | T4N1M0 | iCCA NOS | G3 | S | NA | 72 |
| Asakura^42^ | 2005 | M | 62 | T4N1M0 | iCCA NOS | G2 | S + aK | y | 77 |
| Uenishi^43^ | 2006 | F | 63 | T1aN1M1 | iCCA NOS | G3 | S | n | 84 |
| Kitagawa^44^ | 2008 | NA | NA | NA | IG iCCA | G1 | S | y | 134 |
| Morise^45^ | 2008 | F | 57 | T4N1M0 | iCCA NOS | G2 | S + aK | y | 65 |
| Thomas^46^ | 2008 | NA | NA | T4N0M0 | iCCA NOS | G3 | S | y | 96 |
| Kim^47^ | 2009 | F | 54 | T1bN0M0 | iCCA NOS | G1 | K + S + aK | y | 66* |
| Kamphues^48^ | 2010 | F | 62 | NA | iCCA NOS | NA | S | y | 69 |
| Saiura^49^ | 2011 | M | 67 | T2N1M0 | MF iCCA | G2 | S | y | 130 |
| Saiura^49^ | 2011 | M | 59 | T2N0M0 | MF iCCA | G1 | S + K | y | 79 |
| Saiura^49^ | 2011 | M | 44 | T2N0M0 | MF iCCA | G2 | S | y | 137 |
| Mimatsu^50^ | 2011 | M | 60 | T4N0M1 | MF iCCA | G3 | S + aK | n | 84 |
| Kondo^51^ | 2012 | F | 76 | T4N0M0 | MF iCCA | G2 | S | y | 118 |
| Kobayashi^52^ | 2014 | M | 62 | T3N0M0 | MF iCCA | NA | S + aK | y | 88 |
| Souche^53^ | 2016 | M | 57 | T2N1M0 | iCCA NOS | NA | S | y | 64 |
| Souche^53^ | 2016 | F | 68 | NA | iCCA NOS | NA | S + aK | y | 75 |
| Souche^53^ | 2016 | M | 63 | NA | iCCA NOS | NA | S | y | 113 |
| Souche^53^ | 2016 | M | 47 | NA | iCCA NOS | NA | S + O | y | 107 |
| Souche^53^ | 2016 | M | 39 | NA | iCCA NOS | NA | S + T | y | 194 |
| Tomioku^54^ | 2016 | F | 59 | T1bN0M0 | CholangioloCA | G1 | S + aK | y | 84 |
| Miyazaki^55^ | 2017 | F | 66 | NA | iCCA NOS | NA | S | y | 108 |
| Miyazaki^55^ | 2017 | F | 78 | NA | iCCA NOS | NA | S | y | 108 |
| Miyazaki^55^ | 2017 | M | 60 | NA | iCCA NOS | NA | S | y | 100 |
| Miyazaki^55^ | 2017 | F | 55 | NA | iCCA NOS | NA | S | y | 96 |
| Miyazaki^55^ | 2017 | M | 68 | NA | iCCA NOS | NA | S | y | 64 |
| Si^56^ | 2017 | F | 18 | TxN1M0 | iCCA NOS | G2 | S | y | 66 |
| Motoyama^57^ | 2017 | F | 75 | NA | iCCA NOS | NA | S | y | 85 |
| Shinohara^58^ | 2017 | M | 40 | T1bNxM0 | CholangioloCA | G2 | S + K + O | y | 110 |
| Yoh^59^ | 2018 | M | 64 | T3NxM0 | iCCA NOS | NA | S | y | 94 |
| Yoh^59^ | 2018 | F | 65 | TxN0M1 | iCCA NOS | NA | S | y | 104 |
| Yoh^59^ | 2018 | M | 46 | T2N0M0 | iCCA NOS | NA | S + aK | y | 142 |
| Yoh^59^ | 2018 | M | 70 | T2NxM0 | iCCA NOS | NA | S + aK | y | 86 |
| Yoh^59^ | 2018 | M | 56 | T2N0M0 | iCCA NOS | NA | S | y | 97 |
| Yoh^59^ | 2018 | F | 43 | T1bN0M0 | iCCA NOS | NA | S | y | 380 |
| Yoh^59^ | 2018 | M | 57 | T2N0M0 | iCCA NOS | NA | S | y | 242 |
| Yoh^59^ | 2018 | M | 59 | T2N0M0 | iCCA NOS | NA | S + aK | y | 210 |
| Yoh^59^ | 2018 | M | 69 | TxN0M1 | iCCA NOS | NA | S + aK | y | 181 |
| Yoh^59^ | 2018 | M | 68 | T2N0M0 | iCCA NOS | NA | S + aK | y | 77 |
| Yoh^59^ | 2018 | F | 57 | NA | iCCA NOS | NA | S | y | 191 |
| Yoh^59^ | 2018 | F | 60 | T2N0M0 | iCCA NOS | NA | S + aK | y | 123 |
| Yoh^59^ | 2018 | F | 79 | T2N0M0 | iCCA NOS | NA | S + aK | y | 71 |
| Ohira^60^ | 2018 | M | 40 | T2N0M0 | MF iCCA | G2 | S | y | 79 |
| Ohira^60^ | 2018 | M | 64 | T2N0M0 | MF iCCA | G2 | S + aK | y | 102 |
| Ohira^60^ | 2018 | M | 67 | T2N0M0 | MF iCCA | G1 | S + aK | y | 82 |
| Ohira^60^ | 2018 | F | 70 | T2N0M0 | MF iCCA | G2 | S | y | 68 |
| Bartsch^61^ | 2021 | NA | NA | T2N0M0 | iCCA NOS | NA | S + O | y | 166 |
| Bartsch^61^ | 2021 | NA | NA | T1aN0M0 | iCCA NOS | NA | S + K | y | 154 |
| Bartsch^61^ | 2021 | NA | NA | T2N0M0 | iCCA NOS | NA | S | y | 62 |
| Bartsch^61^ | 2021 | NA | NA | T1bNxM0 | iCCA NOS | NA | S + O | y | 72 |
| Bartsch^61^ | 2021 | NA | NA | T2N0M0 | iCCA NOS | NA | S | y | 69 |
| Bartsch^61^ | 2021 | NA | NA | T2N0M0 | iCCA NOS | NA | S | y | 61 |
| Bartsch^61^ | 2021 | NA | NA | T2N0M0 | iCCA NOS | NA | S + K | y | 61 |
| Abe^62^ | 2023 | M | 69 | T2N1M0 | iCCA NOS | NA | S + K | y | 78 |
| Holzapfel^63^ | 2023 | F | 72 | NA | iCCA NOS | G1 | NA | NA | 152 |
| Holzapfel^63^ | 2023 | M | 57 | NA | iCCA NOS | G2 | NA | NA | 132 |
| Holzapfel^63^ | 2023 | F | 62 | NA | iCCA NOS | G1 | NA | NA | 108 |
| Holzapfel^63^ | 2023 | M | 57 | NA | iCCA NOS | G1 | NA | NA | 172 |
| Holzapfel^63^ | 2023 | M | 64 | NA | iCCA NOS | G2 | NA | NA | 80 |
| Mean values [σ] |  | F 25 (35.7%) M 32 (45.7%)  NA 13 (18.6%) | 60.2 [58.7-61.7] | T1aN0M0 1 (1.5%)  T1aN1M0 1 (1.5%)  T1aN1M1 2 (2.8%)  T1bN0M0 3 (4.2%)  T1bNxM0 2 (2.8%)  T2N0M0 20 (28.6%)  T2N1M0 3 (4.2%)  T2NxM0 1 (1.5%)  T2N1M1 2 (2.8%)  T3N0M0 2 (2.8%)  T3N1M0 1 (1.5%)  T3NxM0 1 (1.5%)  T4N0M0 4 (5.7%)  T4N1M0 3 (4.2%)  T4N0M1 1 (1.5%)  TxN0M1 2 (2.8%)  TxN1M0 1 (1.5%)  NA 20 (28.6%) | iCCA NOS 49 (70%)  MF iCCA 16 (22.9%)  PI iCCA 2 (2.8%)  IG iCCA 1 (1.5%)  cholangioloCA 2 (2.8%) | G1 10 (14.3%)  G2 15 (21.4%)  G3 4 (5.7%)  NA 41 (58.6%) | S 36 (51.4%)  S+aK 18 (25.7%)  S+O 3 (4.2%)  S+K 5 (7.1%)  S + K + O 1 (1.5%)  K+S+aK 1 (1.5%)  S+T 1 (1.5%)  NA 5 (7.1%) | y 56 (80%)  n 7 (10%)  NA 7 (10%) | 108.2 [102.1-114.24]  96† |

Note: Patients whose follow-up exceeds five years (60 months) are considered “long survival.” The reported age is the patient's age at the diagnosis; follow-up is measured in months. TNM is the pathological TNM and is based on the 8^th^ edition of the AJCC classification. In the cases, authors provided TNM parameters according to the 6th or 7th edition of AJCC without expressing the tumor dimension, the number and location of involved lymph nodes, or the presence of metastasis; the authors decided to report “NA” considering the inconsistency among the oldest versions of AJCC.  Follow-up is measured from the surgical resection, except for the case described by Kim et al. (marked with *): the overall follow-up period of 66 months includes 54 months that followed the biopsy diagnosis but preceded the surgical intervention. †: median of the follow up values.

Abbreviations: Recurr.: presence of recurrence, FU: follow-up, NA: not available, M: male patient, MF: mass-forming neoplasm, S: surgery, n: absence of any disease recurrence, F: female patient, NOS: not otherwise specified, G2: moderately differentiated, aK: adjuvant chemotherapy, y: presence of recurrence, G1: well differentiated, MF: mass-forming pattern of growth, PI: periductal-infiltrating pattern of growth, G3: poorly differentiated; IG: intraductal-growth pattern of growth, nK: neoadjuvant therapy, O: other kind of regional treatment like transhepatic arterial chemo-embolization or radiofrequency ablation, K: chemotherapy, T: liver transplantation, CholangioloCA: cholangiolocarcinoma; σ: standard deviation.
